# Clinical Outcomes of Intra-Articular Ozone Injections in Hip Osteoarthritis: A Retrospective Study Comparing Different Injection Frequencies

**DOI:** 10.3390/jcm15020744

**Published:** 2026-01-16

**Authors:** Burcu Ozalp, Argun Pire, Meltem Uyar, Can Eyigor

**Affiliations:** Department of Anesthesiology and Reanimation, Division of Algology, School of Medicine, Ege University, Izmir 35040, Turkey; argunp@gmail.com (A.P.);

**Keywords:** hip osteoarthritis, ozone therapy, intra-articular injection, medical ozone, pain management, WOMAC, injection frequency

## Abstract

**Background:** This retrospective study evaluated the association between the number of intra-articular ozone injection sessions and clinical outcomes in patients with hip osteoarthritis (OA). **Methods:** Data from 54 patients (65 hips) with Tönnis grade 1–2 hip OA treated at a tertiary algology clinic between 2022 and 2024 were analyzed. Patients were categorized into three groups based on the number of ozone sessions received (1, 2, or 3). Pain and functional status were assessed using the Visual Analog Scale (VAS) and the Western Ontario and McMaster Universities Osteoarthritis (WOMAC) index at baseline and at 4, 12, and 24 weeks post-procedure. **Results:** All groups demonstrated significant improvements in VAS and WOMAC scores compared to baseline (*p* < 0.001). Although the three-session group showed more pronounced numerical improvements in both early and late follow-ups, intergroup differences did not consistently reach statistical significance across all time points. **Conclusions:** Intra-articular ozone application is associated with favorable clinical trends in pain reduction and functional recovery. Our findings suggest that a three-session regimen may provide more pronounced clinical improvement compared to fewer sessions. These findings warrant validation through rigorous, randomized controlled trials.

## 1. Introduction

Osteoarthritis (OA) is one of the leading degenerative musculoskeletal diseases affecting quality of life worldwide. According to the 2021 Global Burden of Disease (GBD) report, OA affects approximately 606 million people globally [1], making it a major cause of disability, particularly in individuals over 60 years of age. The hip joint is the second most affected joint after the knee, affecting 12.59% of the population in Europe [2]. OA is characterized by progressive damage to the articular cartilage and structural changes in the whole joint, often involving structural changes in subchondral bone and low-grade chronic synovium inflammation [3,4].

Pain is the main symptom of OA, resulting in significant functional limitations [5]. The primary conservative management strategies include education, exercise, weight loss, and nonsteroidal anti-inflammatory drugs (NSAIDs) [6,7]. However, non-surgical treatment options remain limited, leading to a high reliance on total joint replacement for advanced symptoms and structural damage [8,9,10]. This scarcity of effective non-surgical options emphasizes the need for safe, minimally invasive procedures, such as intra-articular injections, to support early rehabilitation [11].

Intra-articular ozone injection has emerged as a promising treatment due to its proven anti-inflammatory, analgesic, and immunomodulatory effects [12,13]. Ozone modulates inflammation by reducing catabolic chemokines (e.g., MMP, NO) and inflammatory cytokines (e.g., IL-1, TNF-alpha) while stimulating anti-inflammatory cytokines (e.g., IL-4, IL-10) and anabolic factors (e.g., TGF-alpha, IGF-1) [14]. Furthermore, ozone stimulates the production of antioxidant enzymes (e.g., superoxide dismutase) and promotes oxygenation, which relieves tissue edema and ischemia, contributing to its analgesic effect [12,15].

The effectiveness of intra-articular ozone therapy is well-documented, particularly in knee osteoarthritis, where multiple systematic reviews and clinical trials support its safety and benefits in pain reduction and functional capacity [16,17,18,19]. However, the literature concerning ozone therapy specifically in hip osteoarthritis is extremely limited, with published data primarily restricted to case reports or animal studies [20,21]. Given this substantial gap and the absence of standardized protocols, particularly regarding optimal session frequency, our retrospective study was undertaken to assess the short-term clinical outcomes (pain and function) of intra-articular ozone injections in hip osteoarthritis and to explore the clinical response based on the number of injection sessions (1, 2, or 3 sessions).

## 2. Materials and Methods

This study was conducted after the approval of Ethics Committee of Ege University Medical Research (Decision Number: 25-2T/8, Date: 6 February 2025) by retrospectively investigating and recording the data in the hospital registration system of patients who underwent interventional algological procedures for hip pain between 1 January 2022 and 1 December 2024 in Ege University Algology Clinic.

Patients included in the study were required to have been diagnosed with hip osteoarthritis at grade 1 and grade 2 according to the Tönnis classification [22] and to have received intra-articular ozone injection in one or both hip joints. In patients who received bilateral intra-articular injections (n = 11), each joint was treated as a staged procedure with at least a three-month interval between sessions. The oxygen-ozone (O_2_-O_3_) gas mixture was produced using a medical-grade ozone generator (Salutem, Cemil Has Medikal, Izmir, Turkey). Consequently, each joint was initially documented as a separate clinical unit for pain and functional assessment. Therefore, the final dataset contained 65 independent joint observations from 54 subjects. All participants were informed about the study and their consent was obtained.

Patients with previous hip surgery, any intra-articular injection (PRP, steroid, hyaluronic acid, etc.) within the last 3 months, congenital hip dysplasia, trauma and inflammatory diseases such as rheumatoid arthritis were excluded from the study.

**Figure 1:**
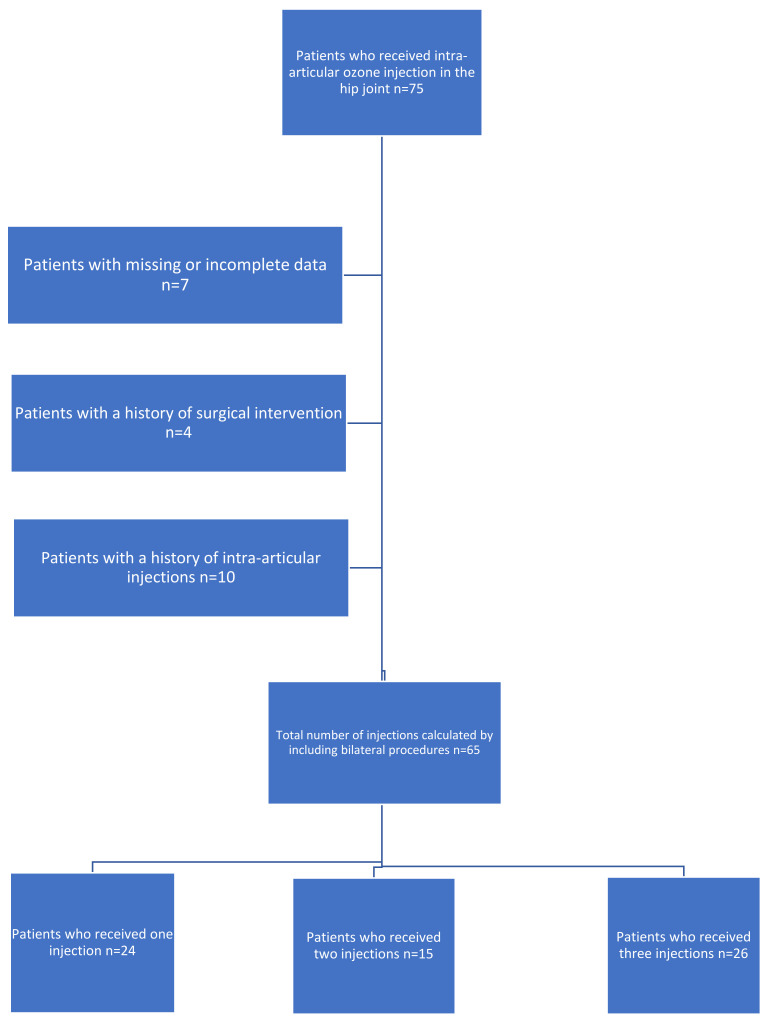
Study flowchart illustrating the selection process and distribution of patients into the three treatment cohorts.

A total of 54 patients (65 hip joints) whose data were available were included in the study. Patients were divided into 3 groups (Group 1: 1 injection, Group 2: 2 injections and Group 3: 3 injections) according to the number of ozone injections received (Figure 1).

The number of ozone applications was determined based on the frequency of clinical application and protocols used in previous knee osteoarthritis studies [18,23].

Procedure: Medical doctors performed the ozone injections in accordance with the Madrid Declaration on Ozone Therapy [24]. Under sterile operating room conditions, providing sedoanalgesia after monitoring the patient in the supine position, a 22G needle (TMT Tıbbi Medikal, İzmir, Turkey) was inserted using a lateral approach into the joint capsule using fluoroscopic guidance after skin anesthesia was produced with local anesthetic. The localization of the needle was confirmed by fluoroscopic imaging after administration of 1 mL of contrast material. Finally, 10 mL of ozone at a dose of 20 µg/mL was injected into each joint and the procedure was completed. After 60 min of monitored observation, the mobilization of the patients was evaluated, and the patients were discharged. The injection was routinely repeated every two weeks in patients who underwent more than one procedure.

Safety and complication monitoring were performed through clinical observation during the procedure and at the 60 min post-operative monitoring period. Furthermore, patients were specifically screened for delayed complications, such as localized infection, persistent pain, hematoma, or neurological symptoms, during the follow-up assessments at 4, 12, and 24 weeks. Any adverse events were recorded in the hospital electronic registry system.

Demographic data of the patients, including age, weight, height and gender as well as VAS and WOMAC scores before and at 4, 12 and 24 weeks after the procedure, were recorded. VAS and WOMAC scores were used for the assessment of pain and activities of daily living. In addition, the medication of the patients before and after the procedure was recorded. For all the patients, the change in VAS and WOMAC scores and the change in analgesic drug use were evaluated at 4, 12 and 24 weeks after ozone injection. Furthermore, the groups determined according to the number of ozone applications were compared in terms of the change in VAS and WOMAC scores and analgesic drug use at 4, 12 and 24 weeks.

VAS: Pain severity was evaluated using the VAS score. The VAS score is a 10 cm line that represents “no pain” on the left (0 cm) of the scale and “worst pain” (10 cm) on the right end.

WOMAC: It involves 24 questions, grouped into 3 subscales. 5 of the questions are related to pain, 2 to stiffness and 17 to physical function (0 = best score, 96 = worst score) [25]. An approved version of this scale in Turkey was used in our evaluation [26].

### Statistical Analysis

Statistical analyses were performed using IBM SPSS Statistics for Windows, Version 25.0 (IBM Corp., Armonk, NY, USA), with the normality of continuous variables verified via the Shapiro–Wilk test and visual inspection of histograms. An a priori power analysis conducted using G*Power 3.1.9.7 indicated that a total sample size of 60 participants was required to achieve a statistical power of 80% (1-\beta = 0.80) with a significance level of alpha = 0.05 and a predicted effect size (Cohen’s f) of 0.33. Continuous data were expressed as mean ± standard deviation (SD), while categorical variables were presented as frequencies and percentages (n, \%). Baseline demographic characteristics and pre-procedural clinical scores across the three injection groups were compared using one-way analysis of variance (ANOVA). Longitudinal changes in Visual Analog Scale (VAS) and Western Ontario and McMaster Universities Osteoarthritis Index (WOMAC) scores from baseline to the 4th, 12th, and 24th weeks were evaluated using the Paired Samples *t*-test for within-group efficacy and one-way ANOVA followed by Tukey’s HSD post hoc test for intergroup dose–response comparisons. Categorical outcomes, including changes in analgesic medication use, were assessed via the Pearson Chi-square and Binomial tests. In all analyses, a *p*-value of <0.05 was considered statistically significant.

## 3. Results

A total of 54 patients (65 hip joints) were enrolled in the study; their baseline characteristics are summarized in Table 1. The cohort had a mean age of 63.66 ± 14.13 years, 164.09 ± 6.29 cm, 71.06 ± 7.85 kg and a mean BMI of 26.42 ± 3 kg/m^2^, with a female predominance (n = 39, 72.2%).

Baseline demographics and clinical parameters—including age, anthropometric measurements, pain duration, and pre-treatment VAS scores—were comparable across the three study groups (*p* > 0.05). However, a significant intergroup difference was observed in baseline WOMAC scores (*p* = 0.023).

Post-procedural evaluations revealed significant improvements in both VAS and WOMAC scores across all follow-up intervals (4, 12, and 24 weeks) compared to baseline (all *p* < 0.001). Mean values and standard deviations for these changes are detailed in Table 2.

Intergroup analysis of score reductions demonstrated significant differences in 4-week VAS (*p* = 0.0054), 24-week VAS (*p* = 0.0461), and 4-week WOMAC (*p* = 0.0130) scores. Overall, the therapeutic efficacy of ozone injection on pain and physical function remained statistically significant for all study groups throughout the 24-week observation period (*p* < 0.05).

**Figure 2:**
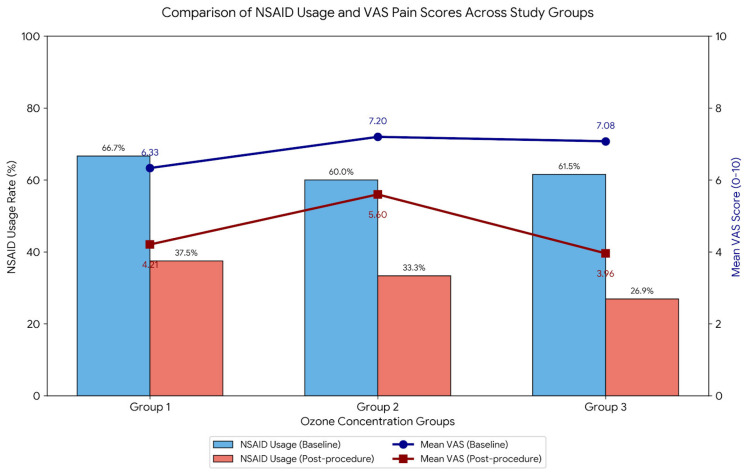
Comparison of NSAID Usage and VAS Pain Scores Across Study Groups.

The change in analgesic medication use was monitored throughout the study period. At baseline, a high percentage of patients in all groups relied on regular NSAID use (Group 1: 66.7%; Group 2: 60.0%; Group 3: 61.5%). Following ozone therapy, a significant reduction in NSAID usage was observed across all cohorts, with the most pronounced decrease in Group 3, reaching 26.9% at the final follow-up. This reduction in medication requirement occurred simultaneously with the improvement in mean VAS scores (Figure 2).

Post hoc pairwise comparisons for 4-week VAS score changes revealed significant differences between Group 1 and Group 2 (*p* = 0.0485), as well as between Group 2 and Group 3 (*p* = 0.0087). Furthermore, Group 3 demonstrated significantly greater VAS score reductions than Group 2 at weeks 12 (*p* = 0.0320) and 24 (*p* = 0.0188). A similar pattern was observed for WOMAC scores at weeks 4 and 24, where Group 3 showed significant improvement over Group 2 (*p* < 0.05). No statistically significant differences in score changes were detected between Group 1 and Group 3 throughout the 24-week follow-up (Table 3).

Regarding safety, no major complications—including intra-articular infection, hematoma, nerve damage, or systemic adverse reactions—were recorded. Minor, self-limiting injection-site discomfort was observed in three patients (n = 3) and did not require intervention.

## 4. Discussion

Our study demonstrated significant improvements in VAS and WOMAC scores at 4, 12, and 24 weeks following intra-articular ozone injections. These results align with previous reports on hip osteonecrosis [20] and animal models of hip osteoarthritis [21], which similarly noted enhanced functional capacity and pain reduction. While ozone therapy is extensively documented for knee osteoarthritis [16,17], clinical data specifically targeting the hip remain limited. Nevertheless, our outcomes are consistent with the established anti-inflammatory, analgesic, and immunomodulatory mechanisms of ozone, which are known to suppress inflammatory mediators and reduce oxidative stress [12].

The observed temporal pattern—maximal benefit at week 4 with sustained significance through week 24—mirrors findings in knee osteoarthritis literature, where efficacy often persists for up to six months [27]. Furthermore, clinical evidence suggesting that 3–5 sessions provide more prolonged benefits in the knee [18,19] supports the rationale for multi-session protocols in the hip joint. Our findings confirm that intra-articular ozone maintains a similar therapeutic trajectory in the hip as observed in more widely studied joints.

Our data indicate a potential relationship between the frequency of injections and the degree of clinical improvement, as the three-session regimen yielded the most pronounced reduction in pain scores and the highest rate of analgesic discontinuation. This suggests that while a single session offers symptomatic relief, a more intensive protocol may exert a more comprehensive anti-inflammatory effect, thereby reducing the systemic drug requirement. This trend parallels findings in knee osteoarthritis literature, where ozone therapy facilitated a significant reduction in analgesic intake [28].

Although the three-injection group demonstrated numerically superior outcomes across all follow-up intervals, the differences compared to the single-injection group did not reach statistical significance. Evidence regarding optimal session frequency remains scarce; however, our findings align with studies on subacromial impingement where multiple injections proved superior [29]. Conversely, the clinical improvement observed in our single-dose group is supported by reports on intradiscal ozone applications, which demonstrate efficacy after a single administration [30,31]. These results contribute valuable insights to the ongoing debate regarding the optimal number of ozone applications in musculoskeletal disorders.

This observation indicates that the clinical response does not follow a classical or linear dose–response relationship. A key factor explaining this pattern is the baseline symptom severity; Group 1 patients presented with significantly milder WOMAC scores at baseline compared to Groups 2 and 3. For these patients with a lower disease burden, a single intervention may have been sufficient to reach clinical satisfaction, explaining the lack of significant difference between one and three sessions in certain outcomes. Furthermore, the inferior outcomes in Group 2 likely reflect a selection bias. This cohort may include an accumulation of ‘non-responders’ who discontinued treatment after two sessions due to insufficient perceived benefit, whereas patients perceiving early relief were more likely to proceed to a third session. Currently, while there are no standardized protocols for ozone therapy and session numbers typically range between 3 and 10, our findings suggest that the clinical course is influenced more by initial joint status and individual response patterns than by a simple cumulative dose effect [23].

One of the strengths of our study is the comparison of groups receiving different numbers of injections, as well as the fact that intra-articular ozone therapy for hip osteoarthritis has not previously been assessed in the literature. Additionally, our follow-up period extends to 24 weeks.

### Limitations

Nevertheless, the study has several limitations; the most significant limitation is the non-comparative, retrospective design, which lacks a control group (e.g., placebo or alternative treatment). Consequently, the observed clinical improvements cannot be definitively attributed solely to the ozone injection, and a potential contribution from the inherent placebo effect associated with any invasive procedure must be acknowledged. Future prospective, randomized controlled trials utilizing a sham control are essential to isolate the specific therapeutic effect of intra-articular ozone.

The retrospective design led to significant baseline differences in WOMAC scores, as patients with more severe symptoms (Groups 2 and 3) were more likely to receive multiple injections. This potential selection bias, compounded by a limited sample size that potentially underpowers inter-group comparisons, necessitates cautious interpretation of differences between ozone protocols. Furthermore, since the WOMAC reflects overall lower limb function, its results may be influenced by unscreened comorbidities in the lumbar spine or knee joints, necessitating a cautious interpretation of the reported functional improvements. Another limitation is that, despite significant clinical gains, the absence of follow-up imaging precludes conclusions regarding structural disease modification; thus, these findings substantiate symptomatic efficacy rather than anatomical joint restoration.

## 5. Conclusions

Intra-articular ozone injection was associated with favorable short-term clinical responses regarding pain and functional limitation in this retrospective cohort of Tönnis Grade 1 and 2 hip osteoarthritis patients. Our exploratory data suggests an association between higher injection frequency—particularly three sessions—and more noticeable clinical improvement.

## Figures and Tables

**Table 1:** Baseline Demographic and Clinical Characteristics of Patients, Compared by Groups.

Characteristic	Group 1 (N = 21)	Group 2 (N = 13)	Group 3 (N = 20)	Total (N = 54)	*p*-Value
Total Hip Count (n)	24	15	26	65	-
Gender (Female/Male, n)	16/5	9/4	14/6	39/15	0.840
Age (Years, Mean ± SD)	62.05 ± 15.81	63.69 ± 16.39	65.42 ± 10.66	63.66 ± 14.13	0.760
Height (cm, Mean ± SD)	163.90 ± 5.56	165.38 ± 8.21	163.42 ± 5.79	164.09 ± 6.29	0.684
Weight (kg, Mean ± SD)	68.24 ± 6.01	72.31 ± 8.99	73.32 ± 8.25	71.06 ± 7.85	0.98
BMI (kg/m^2, Mean ± SD)	25.44 ± 2.41	26.38 ± 2.10	27.54 ± 3.77	26.42 ± 3.00	0.85

Notes: Values are expressed as mean ± standard deviation (SD) for continuous variables and as counts (n) for categorical variables. BMI: Body Mass Index; One-way ANOVA was used for continuous variables (Age, Height, Weight, BMI, Pain Duration), and Chi-square test was used for gender distribution.

**Table 2:** VAS and WOMAC score changes before the application and at 4, 12 and 24 weeks.

		Pre-Application (Mean ± SD)	4th Week Change (Mean ± SD)	12th Week Change (Mean ± SD)	24th Week Change (Mean ± SD)	4th Week Change *p* Value	12th Week Change *p* Value	24th Week Change *p* Value
VAS	Group 1	6.33 ± 1.23	3.12 ± 1.33	2.71 ± 1.60	2.12 ± 1.96	*p* < 0.001	*p* < 0.001	*p* < 0.001
	Group 2	7.20 ± 0.94	1.93 ± 1.94	1.93 ± 1.98	1.60 ± 1.80	*p* < 0.001	*p* < 0.001	*p* < 0.002
	Group 3	7.07 ± 1.01	3.58 ± 1.39	3.42 ± 2.16	3.12 ± 2.03	*p* < 0.001	*p* < 0.001	*p* < 0.001
*p*-value		0.079	0.005 *	0.062	0.046 *			
WOMAC	Group 1	52.7 ± 11.4	21.2±12.4	18.1 ± 14.2	13.62 ± 14.53	*p* < 0.001	*p* < 0.001	*p* < 0.001
	Group 2	61.0 ± 9.4	11.9 ± 15.5	11.6 ± 16.5	9.20 ± 14.1	*p* < 0.005	*p* < 0.008	*p* < 0.012
	Group 3	60.6 ± 9.6	25.5 ± 13.7	21.2 ± 17.0	20.9 ± 18.9	*p* < 0.001	*p* < 0.001	*p* < 0.001
*p*-value		0.023 *	0.013 *	0.184	0.074			

Notes: Values represent mean ± standard deviation (SD). VAS: Visual Analog Scale; WOMAC: Western Ontario and McMaster Universities Osteoarthritis Index. Represent within-group comparison from baseline using Paired Samples *t*-test. Represent comparison between the three injection groups using One-way ANOVA. * Denotes statistically significant difference (*p* < 0.05).

**Table 3:** Pairwise Comparison of Mean Changes in VAS and WOMAC Scores by Number of Injections (Post hoc Analysis).

Comparison	Change Score (Weeks)	Mean Difference (MD)	95% Confidence Interval (CI)	*p*-Value
Group 1 vs. 2	VAS 0–4 Week Change	1.20	[0.07, 2.33]	0.0485
	VAS 0–12 Week Change	0.82	[−0.44, 2.08]	0.1942
	VAS 0–24 Week Change	0.56	[−0.84, 1.95]	0.4232
	WOMAC 0–4 Week Change	8.93	[−1.16, 19.01]	0.0808
	WOMAC 0–12 Week Change	6.11	[−4.67, 16.89]	0.2568
	WOMAC 0–24 Week Change	4.52	[−6.22, 15.27]	0.3975
Group 1 vs. 3	VAS 0–4 Week Change	−0.56	[−1.51, 0.40]	0.2442
	VAS 0–12 Week Change	−0.94	[−2.15, 0.27]	0.1240
	VAS 0–24 Week Change	−1.14	[−2.43, 0.15]	0.0813
	WOMAC 0–4 Week Change	−5.57	[−14.52, 3.39]	0.2163
	WOMAC 0–12 Week Change	−5.22	[−15.29, 4.85]	0.3008
	WOMAC 0–24 Week Change	−9.16	[−20.09, 1.77]	0.0980
Group 2 vs. 3	VAS 0–4 Week Change	−1.76	[−3.00, −0.52]	0.0087
	VAS 0–12 Week Change	−1.76	[−3.23, −0.29]	0.0320
	VAS 0–24 Week Change	−1.70	[−3.09, −0.31]	0.0188
	WOMAC 0–4 Week Change	−14.49	[−25.67, −3.32]	0.0128
	WOMAC 0–12 Week Change	−11.33	[−23.68, 1.02]	0.0708
	WOMAC 0–24 Week Change	−13.68	[−26.50, −0.87]	0.0372

MD: Mean Difference; CI: Confidence Interval; VAS: Visual Analog Scale; WOMAC: Western Ontario and McMaster Universities Osteoarthritis Index. MD values represent the difference in mean change scores between groups. A negative MD indicates a greater reduction (improvement) in the second group compared to the first. Post hoc comparisons were performed using Tukey’s test following a significant ANOVA. Statistical significance is indicated by *p* < 0.05 and 95% CI not crossing zero.

## Data Availability

The data presented in this study are available on request from the corresponding author. The data are not publicly available due to privacy and ethical restrictions.
